# Role of Dexmedetomidine and Clonidine With Hyperbaric Ropivacaine in Subarachnoid Block: A Comprehensive Review

**DOI:** 10.7759/cureus.65798

**Published:** 2024-07-30

**Authors:** Ritika Sharma, Shricha Bhutda, Sakshi Bhutda, Pratiksha Munjewar, Ranjana Sharma

**Affiliations:** 1 Medicine, Jawaharlal Nehru Medical College, Datta Meghe Institute of Higher Education and Research, Wardha, IND; 2 Medical Surgical Nursing, Smt. Radhikabai Meghe Memorial College of Nursing, Datta Meghe Institute of Higher Education and Research, Wardha, IND; 3 Medical Surgical Nursing, Shalinitai Meghe College of Nursing, Datta Meghe Institute of Higher Education and Research, Wardha, IND

**Keywords:** adjuvants, regional anesthesia, clonidine, dexmedetomidine, hyperbaric ropivacaine, subarachnoid block

## Abstract

Subarachnoid block (SAB), a fundamental technique in regional anesthesia, offers efficient anesthesia for various surgical procedures with advantages including rapid onset, reliable anesthesia, and reduced systemic effects compared to general anesthesia. Hyperbaric ropivacaine, a long-acting local anesthetic, has gained popularity due to its favorable pharmacokinetic profile and safety profile. However, to extend the duration and enhance the quality of anesthesia provided by hyperbaric ropivacaine, adjuvants such as dexmedetomidine and clonidine are frequently employed. This comprehensive review explores the roles of dexmedetomidine and clonidine as adjuvants to hyperbaric ropivacaine in SAB. It examines their pharmacological mechanisms, clinical efficacy, safety profiles, and comparative effectiveness in prolonging analgesia and enhancing anesthesia. The review synthesizes evidence from clinical studies to delineate the synergistic effects of these adjuvants, their impact on patient outcomes, and their potential advantages over traditional anesthesia techniques. Through a detailed analysis of current literature and clinical practices, this review aims to provide insights into optimizing the use of dexmedetomidine and clonidine in SAB protocols. It discusses clinical implications, offers recommendations for practice, and identifies future research directions to further enhance the efficacy and safety of SAB using these adjuvants.

## Introduction and background

Subarachnoid block (SAB), also known as spinal anesthesia, is a widely used technique in anesthesiology, providing effective and reliable anesthesia for various surgical procedures [1]. By injecting local anesthetic agents into the cerebrospinal fluid (CSF) in the subarachnoid space, SAB produces a rapid onset of profound sensory and motor blockade. This technique is favored for its simplicity, cost-effectiveness, and ability to provide excellent intraoperative conditions while maintaining the patient’s consciousness and ability to cooperate [2]. SAB is particularly advantageous in lower abdominal, pelvic, and lower extremity surgeries. It reduces the need for general anesthesia, minimizing associated risks such as postoperative nausea and vomiting, respiratory complications, and longer recovery times. Additionally, SAB has a lower incidence of thromboembolic events and is beneficial for patients with certain comorbidities, where general anesthesia poses a higher risk [3].

Hyperbaric ropivacaine is a long-acting amide local anesthetic, structurally related to bupivacaine and mepivacaine, but with a superior safety profile. It is formulated to be denser than CSF, allowing it to settle in the subarachnoid space, thus ensuring a more predictable and controlled spread of anesthesia [4]. The pharmacological properties of ropivacaine, including its lower lipid solubility and partial vasoconstrictive effect, result in prolonged analgesia with a reduced risk of cardiotoxicity and neurotoxicity compared to other local anesthetics. Ropivacaine's pharmacokinetics involve slow absorption from the injection site, extensive protein binding, and hepatic metabolism, making it suitable for prolonged anesthesia [5]. Its favorable sensory-motor differential blockade allows for excellent pain control with less motor impairment, which is particularly beneficial for postoperative recovery and early mobilization [6].

Despite the advantages of SAB, the duration and quality of anesthesia provided by local anesthetics alone may sometimes be insufficient for lengthy or highly painful procedures. Adjuvants are therefore used to enhance and prolong the effects of local anesthetics, reducing the need for additional doses and minimizing potential side effects [7]. Adjuvants such as opioids, alpha-2 adrenergic agonists (e.g., dexmedetomidine and clonidine), and others can be added to the local anesthetic solution to improve the quality of anesthesia and analgesia. Dexmedetomidine and clonidine have garnered significant attention due to their synergistic effects with local anesthetics. These agents act on spinal cord receptors to enhance analgesia, prolong the duration of action, and provide sedation without significant respiratory depression. These adjuvants can improve patient outcomes, provide greater intraoperative comfort, and improve postoperative pain management [8].

The primary objective of this comprehensive review is to elucidate the roles of dexmedetomidine and clonidine as adjuvants to hyperbaric ropivacaine in SAB. This review analyzes the pharmacological profiles of hyperbaric ropivacaine, dexmedetomidine, and clonidine. Additionally, it seeks to evaluate the clinical efficacy and safety of using dexmedetomidine and clonidine with hyperbaric ropivacaine. The review will compare the individual and combined effects of these adjuvants on the quality and duration of anesthesia and analgesia and discuss the mechanisms through which these agents enhance the anesthetic effects of hyperbaric ropivacaine. Furthermore, it will provide clinical guidelines and recommendations for using dexmedetomidine and clonidine in SAB and identify areas for future research and potential new adjuvants for enhancing SAB. Through this review, we hope to provide a comprehensive understanding of how dexmedetomidine and clonidine can be effectively integrated into SAB protocols, thereby optimizing patient care and expanding the utility of this vital anesthetic technique.

## Review

Hyperbaric ropivacaine in subarachnoid block

Pharmacology and Mechanism of Action

Dexmedetomidine is a highly selective α2-adrenoceptor agonist with a distinct mechanism of action differing from other sedatives like clonidine. It selectively binds to α2-adrenoceptors in the central nervous system (CNS), inhibiting neuronal firing and reducing sympathetic activity [9]. This binding induces sedation, anxiolysis, and analgesia by diminishing norepinephrine release and inhibiting sympathetic activity, lowering blood pressure and heart rate. Activation of α2-adrenoceptors in the locus coeruleus produces a state of unconsciousness akin to natural sleep, with patients easily arousable and cooperative. Dexmedetomidine lacks direct effects on the heart but can elicit a biphasic cardiovascular response, including transient hypertension, bradycardia, and hypotension [10]. Dexmedetomidine's analgesic properties are notable as well. It hampers pain transmission by curtailing norepinephrine release at the spinal level, halting the propagation of pain signals. This analgesic effect is dose-dependent, plateauing beyond doses exceeding 0.5 μg/kg. This characteristic indicates a predictable and safe analgesic profile. These analgesic attributes render dexmedetomidine valuable for pain management, particularly in scenarios where respiratory depression is a concern [9]. Administered intravenously, dexmedetomidine swiftly distributes throughout the body, boasting a volume of distribution (VD) of 118 L and a half-life of approximately 6 minutes. It exhibits high protein binding (94%) and a context-sensitive half-life varying from 4 minutes for a 10-minute infusion to 250 minutes for an 8-hour infusion. Primarily renally excreted (95%) with a minor fecal component (4%), its pharmacokinetic profile allows for rapid onset and relatively short duration, making it suitable for short-term sedation and analgesia [11].

Benefits and Limitations

Dexmedetomidine offers several clinical advantages. It provides a unique sedative response, allowing patients to remain cooperative and responsive when stimulated, which is beneficial for procedures requiring patient cooperation [12]. Moreover, it has a lower propensity to cause delirium compared to other sedatives and may even prevent its occurrence. Dexmedetomidine also possesses analgesic properties, though less potent than other analgesics, making it a valuable adjunct in pain management. It can reduce the requirement for opioids and other analgesics, contributing to opioid-sparing effects. Additionally, dexmedetomidine's sympatholytic effects help stabilize hemodynamics during surgery, thereby lowering the incidence of tachycardia and hypertension. It also assists in managing perioperative stress responses, including fluctuations in blood pressure and heart rate [13]. However, dexmedetomidine has limitations. It can lead to bradycardia and hypotension, particularly at higher doses or when combined with other anesthetics, which can be problematic in patients with pre-existing cardiovascular conditions. Additionally, dexmedetomidine may induce systemic and pulmonary hypertension due to its direct effects on peripheral blood vessels [14]. While it minimally affects respiratory drive, it can contribute to airway obstruction when used concurrently with other anesthetics, which poses risks, especially in patients with respiratory issues. Furthermore, dexmedetomidine may cause adverse events such as bradycardia, hypotension, and even cardiac arrest, particularly during or shortly after a loading infusion. It can also prolong recovery times and sedation duration, which may present challenges in certain clinical contexts [15].

Clinical Applications and Outcomes

**Figure 1:**
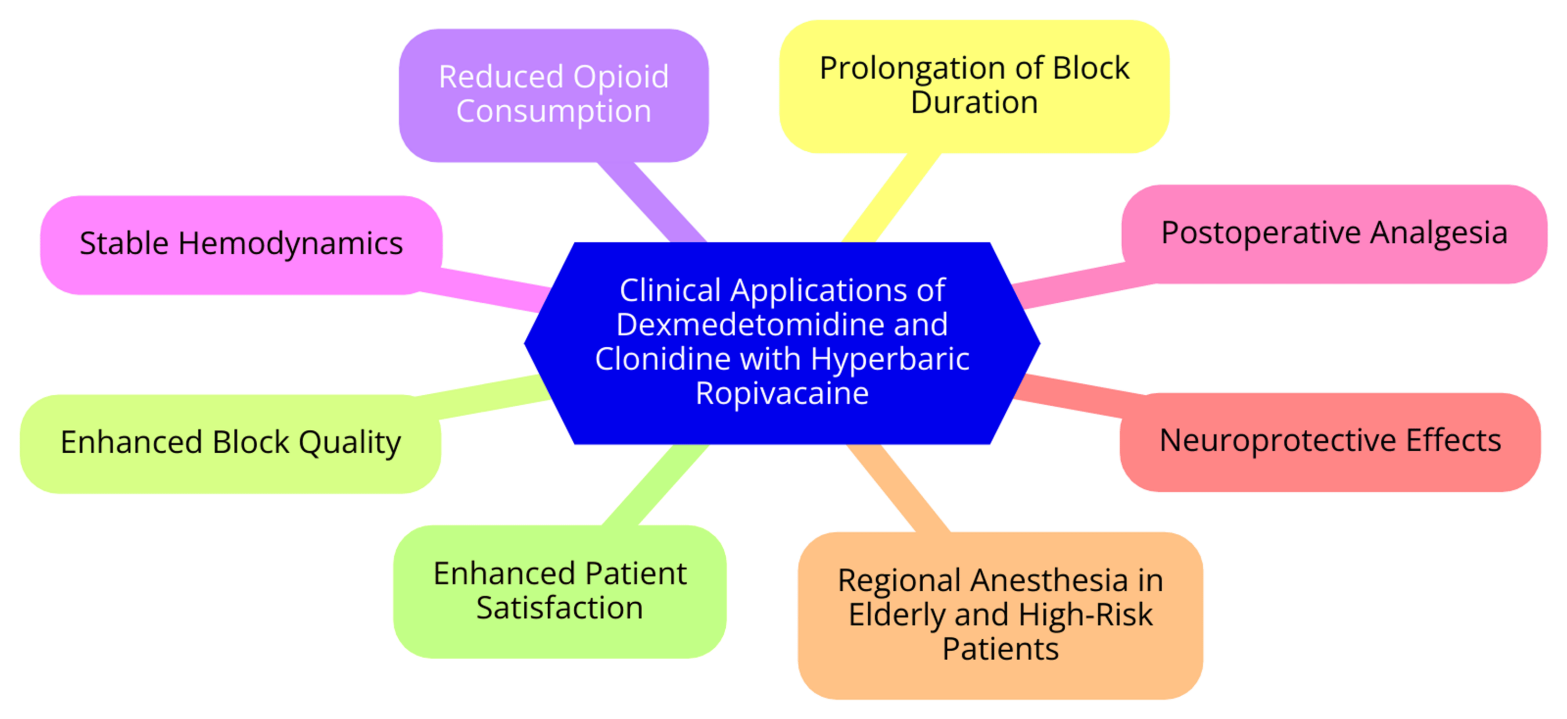
Clinical applications of dexmedetomidine and clonidine with hyperbaric ropivacaine in the subarachnoid block Image credit: Pratiksha Munjewar

Hyperbaric ropivacaine, a local anesthetic increasingly utilized in subarachnoid blocks for spinal anesthesia, offers distinct advantages and disadvantages compared to hyperbaric bupivacaine [16]. One notable advantage is its improved hemodynamic profile. Studies indicate that combining hyperbaric and hypobaric ropivacaine significantly reduces the incidence of hypotension and related complications compared to using hyperbaric ropivacaine alone. This benefit is particularly crucial in procedures requiring stable hemodynamics, such as those involving elderly or high-risk patients [17]. In terms of efficacy and duration, hyperbaric ropivacaine reliably provides spinal anesthesia but generally has a shorter duration compared to hyperbaric bupivacaine. Hyperbaric bupivacaine offers a quicker onset of sensory block and longer-lasting analgesia, making it preferable for extended surgical procedures. Conversely, hyperbaric ropivacaine's shorter recovery profile makes it suitable for intermediate-duration surgeries [18]. Safety is another critical factor in choosing between these local anesthetics. Ropivacaine is generally considered safer than bupivacaine due to its lower risk of cardiotoxicity and central nervous system (CNS) toxicity. This safety profile enhances the attractiveness of hyperbaric ropivacaine, especially in scenarios where systemic toxicity is a concern [19]. Clinical applications of dexmedetomidine and clonidine with hyperbaric ropivacaine in the subarachnoid block are shown in Figure 1.

Dexmedetomidine as an adjuvant

Pharmacology and Mechanism of Action

Dexmedetomidine is a highly selective α2-adrenergic agonist known for its unique pharmacological profile and mechanism of action, distinct from other sedative agents like clonidine. By activating α2-adrenoceptors in the brain and spinal cord, dexmedetomidine inhibits neuronal firing, resulting in a range of effects, including hypotension, bradycardia, sedation, and analgesia [9]. This activation also reduces salivation, secretion, and gastrointestinal motility, contracts vascular and smooth muscle, inhibits renin release, increases glomerular filtration and sodium/water excretion in the kidney, lowers intraocular pressure, and decreases insulin release from the pancreas. The presynaptic activation of α2-adrenoceptors blocks the release of norepinephrine, thereby halting the transmission of pain signals. Postsynaptic activation in the CNS inhibits sympathetic activity, leading to decreased blood pressure and heart rate. These combined effects provide effective analgesia, sedation, and anxiolysis, making dexmedetomidine versatile in sedation and analgesia practices [20]. Notably, dexmedetomidine allows patients to remain easily arousable, distinguishing it from other sedatives. In clinical settings, dexmedetomidine is commonly used in intensive care units for its stable hemodynamic profile and minimal respiratory depression. It serves as an adjunct in anesthesia, particularly beneficial for obese patients at risk of postoperative respiratory complications due to its opioid-sparing effects and minimal impact on respiration. Dexmedetomidine is approved for procedural sedation and has shown efficacy in various off-label uses, such as pediatric sedation and as an adjuvant to local analgesia techniques. Dexmedetomidine's pharmacology and mechanism make it a valuable agent for sedation, analgesia, and anesthesia, combining sedative, anxiolytic, and sympatholytic effects with minimal respiratory depression and stable hemodynamics [11].

Benefits of Dexmedetomidine in SAB

Dexmedetomidine offers several advantages when used as an adjuvant in subarachnoid block (SAB). One key benefit is its ability to prolong the duration of sensory and motor blocks. When combined with intrathecal bupivacaine, dexmedetomidine extends the time to sensory regression to the L1 dermatome and facilitates complete motor recovery compared to bupivacaine alone. Studies have demonstrated that intrathecal dexmedetomidine (5 μg) enhances the analgesic effects of hyperbaric bupivacaine by up to 31% in spinal anesthesia [21]. Another significant advantage of dexmedetomidine is its capacity to provide sedation while maintaining hemodynamic stability without causing significant side effects. This characteristic makes patients more alert and responsive to verbal commands during sedation, which is particularly advantageous in certain surgical settings [11]. Dexmedetomidine also contributes to reducing the required dose of intrathecal local anesthetics. By decreasing the ED95 (effective dose in 95% of patients) of spinal hyperbaric bupivacaine, dexmedetomidine helps mitigate spinal-induced hypotension associated with high doses of intrathecal local anesthetics [22]. Furthermore, dexmedetomidine has been found to extend the duration of postoperative analgesia when added as an adjuvant to local anesthetics in both central and peripheral nerve blocks. Compared to controls, intrathecal dexmedetomidine enhances the duration of analgesia, providing prolonged pain relief [23].

Clinical Studies and Outcomes With Hyperbaric Ropivacaine

The use of hyperbaric ropivacaine with dexmedetomidine and clonidine as adjuvants in the subarachnoid block has been extensively studied, revealing significant enhancements in sensory and motor block durations compared to ropivacaine alone [24]. Research indicates that adding dexmedetomidine (3 μg) or clonidine (30 μg) to intrathecal hyperbaric ropivacaine notably prolongs sensory regression to the S1 segment and motor block regression to Bromage 0. Specifically, sensory regression times were 303 ± 75 minutes with dexmedetomidine, 272 ± 38 minutes with clonidine, and 190 ± 48 minutes with ropivacaine alone (p<0.001). Similarly, motor block regression times were 250 ± 76 minutes with dexmedetomidine, 216 ± 35 minutes with clonidine, and 163 ± 47 minutes with ropivacaine alone (p<0.001) [25]. Interestingly, the dexmedetomidine and clonidine groups' onset times for sensory and motor blocks were comparable. In terms of hemodynamic effects and sedation, both dexmedetomidine and clonidine, when added to intrathecal hyperbaric ropivacaine, did not induce significant changes in mean arterial pressure, heart rate, or sedation levels compared to ropivacaine alone, intraoperatively or postoperatively. This indicates that these adjuvants can be safely used without compromising patient stability [26]. A direct comparison between dexmedetomidine (5 μg) and clonidine (50 μg) added to intrathecal hyperbaric ropivacaine showed significantly prolonged sensory and motor blockade durations compared to ropivacaine alone. However, dexmedetomidine demonstrated greater prolongation of these parameters than clonidine [26].

Side Effects and Safety Profile

Dexmedetomidine, while effective, can lead to a range of side effects, both common and less common, which should be monitored closely during its use [27]. Common side effects include blurred vision, chest discomfort, confusion, coughing, dizziness, headaches, irregular breathing, nervousness, pale or bluish skin and lips, pounding in the ears, changes in heartbeat rhythm, sweating, breathing difficulties, and unusual fatigue or weakness. Less common side effects may involve bleeding gums, bloody urine, coughing up blood, drowsiness, dry mouth, fever, flushed skin, increased thirst and hunger, increased urination, muscle cramps, nausea, nosebleeds, numbness or tingling, prolonged bleeding from cuts, seizures, swelling of extremities or face, tremors, unexplained weight changes, and vomiting [27]. Certain side effects have an unknown incidence rate, such as chest tightness, confusion, frequent urination, hallucinations, increased urine output, irritability, low blood pressure, pain in arms, jaw, back, or neck, restlessness, hallucinations, shakiness, drowsiness, slow response, slurred speech, stomach pain, cardiac arrest, unconsciousness, unusual excitement or restlessness, weakness or heaviness in legs, and yellowing of eyes or skin [28]. Cardiovascular effects are frequent with dexmedetomidine use, including hypotension (56%), bradycardia (42%), systolic hypertension (28%), requiring intervention for hypotension (28%), tachycardia (25%), and requiring intervention for hypertension (25%) [9]. Other significant safety considerations include potential withdrawal symptoms after prolonged use and tachycardia and hypertension following discontinuation. Dexmedetomidine should be used cautiously in patients with hepatic impairment and certain cardiac conditions. Prolonged exposure may lead to tolerance and tachyphylaxis. Drug interactions are also a concern, particularly with vasodilators, negative chronotropic agents, anesthetics, sedatives, hypnotics, and opioids. Special considerations are necessary during pregnancy and lactation [29].

Clonidine as an adjuvant

Pharmacology and Mechanism of Action

Dexmedetomidine, a highly selective α2-adrenoceptor agonist, exerts its effects primarily by activating central pre- and post-synaptic α2-receptors in the brain's locus coeruleus [9]. This activation inhibits the release of norepinephrine, thereby interrupting pain signal transmission and inducing analgesia, sedation, and anxiolysis. In addition to its central actions, dexmedetomidine influences peripheral physiology, causing vasoconstriction, vasodilatation, and reflex bradycardia through α2-receptor activation. The pharmacokinetics of dexmedetomidine are characterized by rapid distribution and a short initial half-life of approximately 6 minutes. It undergoes extensive hepatic metabolism via glucuronidation and hydroxylation, resulting in the formation of inactive metabolites. Dexmedetomidine exhibits significant variability in its pharmacokinetics among individuals, particularly in settings like intensive care, where factors such as body size, hepatic function, plasma albumin levels, and cardiac output can markedly influence its effects [10]. In contrast, clonidine, another α2-adrenoceptor agonist, is less selective than dexmedetomidine, with an α2:α1 ratio of around 220:1. Like dexmedetomidine, clonidine acts centrally to produce sedation, analgesia, and sympatholysis by stimulating α2-receptors. It also shares peripheral effects such as vasoconstriction, vasodilatation, and reflex bradycardia [20]. However, the differing selectivity ratios and pharmacokinetic profiles between dexmedetomidine and clonidine contribute to their distinct clinical applications and potential side effects.

Benefits of Clonidine in SAB

Clonidine, an alpha-2 adrenergic agonist, has been extensively investigated for its role as an adjuvant in subarachnoid block (SAB), particularly when combined with local anesthetics like bupivacaine or ropivacaine [30]. Adding clonidine in low doses (15-30 μg) to intrathecal local anesthetics has shown several benefits in clinical studies. One of the primary advantages of using clonidine as an adjuvant is its ability to prolong sensory and motor blockade duration compared to local anesthetics alone. For instance, research has demonstrated that adding clonidine (50 μg) to bupivacaine significantly extends the duration of motor blockade, with one study reporting a mean duration of 280.80 minutes compared to 183.60 minutes with bupivacaine alone [30]. Similarly, clonidine enhances postoperative analgesia duration; studies have shown a prolonged analgesic effect of up to 551.06 minutes when clonidine is added to bupivacaine, compared to 254.80 minutes with bupivacaine alone [30]. This extended pain relief can reduce the need for additional pain medications in the postoperative period. Additionally, while evidence on onset time is less conclusive, some studies suggest that clonidine may expedite the onset of sensory and motor blockade when used as an adjuvant to intrathecal local anesthetics [30]. However, the use of clonidine in SAB does come with potential drawbacks. It can induce hemodynamic side effects such as hypotension and bradycardia, which require careful clinical management. The incidence of these side effects tends to correlate with dosage, with higher doses (e.g., 30 μg) potentially causing more pronounced effects [30]. Therefore, clinicians must weigh these benefits against the risks and monitor patients closely when using clonidine as an adjuvant in SAB.

Clinical Studies and Outcomes With Hyperbaric Ropivacaine

Hyperbaric ropivacaine has been extensively studied as a spinal anesthetic agent, often compared to the more commonly used hyperbaric bupivacaine. While hyperbaric ropivacaine does exhibit a slower onset of sensory and motor blockade compared to hyperbaric bupivacaine, it offers significant advantages in terms of hemodynamic stability. Patients administered hyperbaric ropivacaine tend to experience less hypotension, making it a preferred choice, especially in elderly or cardiovascularly compromised individuals [16]. Despite its shorter duration of sensory and motor blockade compared to bupivacaine, hyperbaric ropivacaine still provides adequate anesthesia for lower abdominal, perineal, and lower limb surgeries. Its motor-sparing effect allows quicker regression of motor blockade and earlier patient ambulation, which aligns well with enhanced recovery after surgery (ERAS) protocols [18]. Researchers have explored the use of adjuvants to enhance the clinical outcomes of hyperbaric ropivacaine. For example, adding intrathecal magnesium sulfate to hyperbaric ropivacaine has been found to significantly prolong sensory blockade and postoperative analgesia without extending the duration of motor blockade. This approach effectively optimizes hyperbaric ropivacaine's anesthetic and analgesic properties, offering a promising strategy for improving patient comfort and recovery post-surgery [31].

Side Effects and Safety Profile

Clonidine, an alpha-2 adrenergic agonist commonly used as an adjuvant in regional anesthesia, offers significant benefits in prolonging sensory and motor blockade and enhancing postoperative analgesia. However, its use necessitates awareness of potential side effects ranging from mild to severe. Common side effects of clonidine include drowsiness, dizziness, dry mouth, constipation, and disturbances in sleep patterns [32]. More serious adverse effects may include hypotension (low blood pressure), light-headedness, fainting, bradycardia (slow heart rate), and rebound hypertension (high blood pressure) if clonidine is abruptly discontinued [32]. The severity and likelihood of these side effects generally increase with higher doses of clonidine and may be more pronounced in elderly patients or those with pre-existing cardiovascular conditions [32]. Clonidine's interactions with other medications affecting the cardiovascular or central nervous systems can further heighten these risks [33]. Close monitoring of patients receiving clonidine is crucial, especially during treatment initiation, dosage adjustments, or when combined with other medications. Monitoring should focus on detecting hypotension, bradycardia, and sedation, with appropriate adjustments or supportive measures implemented as needed [33]. Patients should also be advised against engaging in activities requiring alertness until they understand their response to clonidine. To mitigate the risks associated with discontinuation, clonidine should be tapered off gradually under medical supervision to prevent withdrawal symptoms and sudden increases in blood pressure [34]. This approach ensures a safer transition and minimizes potential adverse effects.

Comparison of dexmedetomidine and clonidine

Efficacy in Prolonging Analgesia and Anesthesia

The addition of dexmedetomidine or clonidine to intrathecal ropivacaine or bupivacaine has been studied extensively, highlighting their ability to prolong sensory and motor blockade durations and enhance postoperative analgesia. When dexmedetomidine (3 μg) or clonidine (30 μg) is added to intrathecal hyperbaric bupivacaine, it results in faster onset and longer duration of sensory and motor blockade compared to bupivacaine alone, with dexmedetomidine typically producing more pronounced effects [35]. Both dexmedetomidine and clonidine, when added to intrathecal bupivacaine, extend the duration of postoperative analgesia. Dexmedetomidine delays the time to the first postoperative analgesic request (243.35 ± 56.82 min) more than clonidine (190.93 ± 42.38 min) and placebo (140.75 ± 28.52 min) [35]. Similarly, adding low-dose clonidine (15-30 μg) to intrathecal hyperbaric ropivacaine significantly prolongs sensory and motor blockade durations and enhances postoperative analgesia compared to ropivacaine alone [36]. While dexmedetomidine generally produces a more pronounced prolongation of sensory and motor blockade than clonidine, clonidine is noted for its cost-effectiveness as an intrathecal adjuvant. Dexmedetomidine is approximately five times more expensive than clonidine, making clonidine a preferred choice in settings where cost considerations are paramount [37].

Effects on Hemodynamics

Dexmedetomidine exerts a stabilizing effect on hemodynamics when used as an intrathecal adjuvant. It reduces mean arterial pressure and heart rate by inhibiting sympathetic nerve activity and decreasing catecholamine release. Dexmedetomidine maintains hemodynamic stability without causing significant sedation or adverse effects like hypotension and bradycardia, especially when administered within appropriate doses (0.5-0.8 μg/kg) [9]. In contrast, the addition of clonidine (15-30 μg) to intrathecal ropivacaine tends to increase the incidence of intraoperative hypotension and bradycardia. While these hemodynamic effects can typically be managed with standard clinical measures, they are more pronounced than the favorable hemodynamic profile observed with dexmedetomidine as an intrathecal adjuvant [35]. Therefore, dexmedetomidine may be preferred in situations where maintaining stable hemodynamics is critical, such as in high-risk patients or those undergoing prolonged surgical procedures.

Side Effect Profiles

Both dexmedetomidine and clonidine are effective intrathecal adjuvants when combined with hyperbaric bupivacaine or ropivacaine, enhancing sensory and motor blockade durations and prolonging postoperative analgesia. Dexmedetomidine typically demonstrates a more pronounced sensory and motor blockade prolongation than clonidine [26]. Specifically, dexmedetomidine delays the time to the first postoperative analgesic request to a greater extent (243.35 ± 56.82 min) compared to clonidine (190.93 ± 42.38 min) and placebo (140.75 ± 28.52 min) [26]. In terms of hemodynamic effects, clonidine (15-30 μg) added to intrathecal ropivacaine has been associated with an increased incidence of intraoperative hypotension and bradycardia. Conversely, dexmedetomidine and clonidine as intrathecal adjuvants with bupivacaine do not typically induce significant sedation either intraoperatively or postoperatively [35]. However, dexmedetomidine has a higher incidence of hypotension (56%) and bradycardia (42%), compared to clonidine when used intrathecally. While both drugs share common side effects like hypotension and bradycardia, clonidine is more likely to cause clinically significant hypotension and bradycardia, requiring intervention when added to intrathecal ropivacaine [35]. Despite their similar efficacy as intrathecal adjuvants, dexmedetomidine is approximately five times more costly than clonidine, making clonidine a more cost-effective option [35]. Serious side effects of dexmedetomidine can include respiratory depression, irregular heartbeats, allergic reactions, agitation, confusion, and muscle weakness. However, neither dexmedetomidine nor clonidine typically cause significant sedation when used in this manner [9].

Patient Outcomes and Satisfaction

Both dexmedetomidine and clonidine have been extensively studied as adjuvants to intrathecal local anesthetics like hyperbaric bupivacaine or ropivacaine. When added to these spinal anesthetics, both drugs are effective in prolonging the onset and duration of sensory and motor blockade, although dexmedetomidine tends to produce a more pronounced effect [38]. Specifically, the addition of dexmedetomidine (3-5 μg) or clonidine (30 μg) to intrathecal hyperbaric bupivacaine or ropivacaine results in faster onset and longer duration of sensory and motor blockade compared to the local anesthetic alone [37]. In terms of postoperative analgesia, both dexmedetomidine, and clonidine, when added to intrathecal bupivacaine, extend the duration of pain relief after surgery. Dexmedetomidine demonstrates superior efficacy in this regard, significantly increasing the time to the first postoperative analgesic request (243 minutes) compared to clonidine (191 minutes) and placebo (141 minutes) [37].

Regarding hemodynamic effects, the addition of clonidine (15-30 μg) to intrathecal ropivacaine has been associated with a higher incidence of intraoperative hypotension and bradycardia. In contrast, neither dexmedetomidine nor clonidine as intrathecal adjuvants with bupivacaine appears to cause significant sedation during the intraoperative or postoperative period [35]. Despite their comparable efficacy as intrathecal adjuvants, dexmedetomidine is approximately five times more costly than clonidine. Therefore, clonidine may be considered a more cost-effective option when used as an adjunct to intrathecal bupivacaine or ropivacaine despite its slightly less pronounced effects compared to dexmedetomidine [26].

Clinical applications and guidelines

Indications for Using Dexmedetomidine and Clonidine With Hyperbaric Ropivacaine

Adding low-dose clonidine (15-30 μg) or dexmedetomidine (3-5 μg) to intrathecal hyperbaric ropivacaine is a valuable clinical approach to significantly extend the duration of sensory and motor blockade. Studies indicate that these alpha-2 agonist adjuvants can lengthen sensory blockade by 40-80 minutes and motor blockade by 50-100 minutes, particularly beneficial in procedures requiring prolonged spinal anesthesia [35]. In addition to enhancing block duration, dexmedetomidine and clonidine improve postoperative analgesia compared to ropivacaine alone, with dexmedetomidine generally demonstrating superior pain relief outcomes [39]. However, using these adjuvants also increases the incidence of intraoperative hemodynamic effects. The occurrence of hypotension (up to 83%) and bradycardia (up to 23%) is more frequent compared to ropivacaine alone, necessitating careful clinical management and monitoring [8]. Considering cost-effectiveness, clonidine, despite being less potent than dexmedetomidine, is approximately five times cheaper. This cost advantage makes clonidine a practical choice when aiming to prolong spinal anesthesia with ropivacaine [26].

Recommended Protocols for Different Surgical Procedures

Preoperative protocols should incorporate considerations for potential COVID-19 infection in all patients requiring emergency surgery. Hospitals should establish dedicated transport routes to the operating room and prioritize aerosol-generating procedures in negative pressure rooms whenever possible. Without negative pressure rooms, these procedures, including induction and recovery, should be conducted within the operating room [40]. Intraoperative protocols are aimed at creating a controlled environment that minimizes exposure risks. Ideally, negative-pressure operating rooms should be used; alternatively, positive-pressure operating rooms with sufficient air exchanges (20-25 per hour) can be utilized. Measures should be taken to limit the number of personnel, equipment, and traffic entering and exiting the operating room. A designated drop-off area outside the OR and a dedicated runner for retrieving supplies help reduce unnecessary movement within the surgical environment. Energy devices should be operated at the lowest effective setting, and smoke generated during procedures should be promptly evacuated using suction devices [41]. Postoperative protocols emphasize allowing adequate time between procedures for settling aerosolized viral particles. Routine cleaning and disinfection protocols should be followed rigorously, employing Environmental Protection Agency (EPA)-approved disinfectants known to be effective against SARS-CoV-2. These comprehensive protocols, endorsed by major healthcare organizations and surgical societies, are designed to optimize safety across the perioperative setting during the COVID-19 pandemic [42].

Patient Selection Criteria and Considerations

Patient selection criteria and considerations for transitioning from dexmedetomidine to clonidine for sedation management involve several key factors. Age and health status significantly influence this decision, with clonidine generally preferred for younger patients due to its lower risk of adverse effects and better tolerability compared to dexmedetomidine. Conversely, dexmedetomidine is often favored for elderly patients because of its higher alpha-2/alpha-1 receptor selectivity, which can be beneficial for this demographic [43]. The duration of sedation is another critical consideration. While dexmedetomidine is approved for up to 24 hours of sedation, it is frequently used for longer periods of time. Clonidine offers advantages for long-term sedation since it can be administered orally or transdermally, facilitating the transition out of the ICU and potentially reducing costs [44]. Hemodynamic effects are a crucial factor when choosing between dexmedetomidine and clonidine. Both medications can cause hypotension and bradycardia, but clonidine generally has a lower incidence of these effects compared to dexmedetomidine. Additionally, clonidine is considered to have a slightly better cardiovascular safety profile, particularly in critically ill patients after cardiac surgery [45]. Withdrawal symptoms are another important consideration. Discontinuation of dexmedetomidine can lead to withdrawal symptoms such as tachycardia, hypertension, and agitation. Clonidine is often used to help mitigate these symptoms during the weaning process. Although clonidine withdrawal syndrome is less well-defined, it can include symptoms like hypertension and agitation [46]. Cost and administration are also crucial factors. Clonidine is a more cost-effective option compared to dexmedetomidine, making it a practical choice for prolonged sedation. Furthermore, clonidine’s oral or transdermal administration can facilitate the transition out of the ICU and reduce costs [47]. Finally, clinical management considerations, such as monitoring and tapering, are vital. Both drugs require close monitoring for hemodynamic effects and potential withdrawal symptoms, but clonidine may be easier to manage due to its lower incidence of adverse effects. Clonidine is often used in conjunction with dexmedetomidine tapering to minimize withdrawal symptoms and ensure a smooth transition [48].

## Conclusions

In conclusion, integrating dexmedetomidine and clonidine as adjuvants to hyperbaric ropivacaine in subarachnoid block (SAB) presents significant advancements in regional anesthesia. Both dexmedetomidine and clonidine enhance the analgesic and anesthetic effects of hyperbaric ropivacaine, leading to a prolonged duration of action, improved quality of anesthesia, and better patient outcomes. These adjuvants provide additional benefits, such as sedation and anxiolysis, with minimal respiratory depression, making them particularly valuable in various surgical settings. The comparative analysis reveals that while both agents are effective, their unique pharmacological profiles offer different advantages, allowing for tailored anesthetic management based on individual patient needs and surgical requirements. The evidence supports the use of these adjuvants for their efficacy and safety, making them integral components in modern SAB practices. Future research should continue to explore optimal dosing regimens, combinations, and potential new adjuvants to further enhance the efficacy and safety of SAB, ultimately improving patient care and surgical outcomes.
